# Nutritional, Rheological, and Functional Assessment in the Development of Bread Using Chestnut and Rosehip-Fortified Wheat Flour

**DOI:** 10.3390/foods14193343

**Published:** 2025-09-26

**Authors:** Ioana-Alina Pop, Sylvestre Dossa, Daniela Stoin, Christine Neagu, Diana Moigradean, Ersilia Alexa, Mariana-Atena Poiana

**Affiliations:** Faculty of Food Engineering, University of Life Sciences “King Mihai I” from Timisoara, Calea Aradului No. 119, 300645 Timisoara, Romania; alina.pop@usvt.ro (I.-A.P.); dossasylvestre@usvt.ro (S.D.); danielastoin@usvt.ro (D.S.); christine.neagu@usvt.ro (C.N.); dianamoigradean@usvt.ro (D.M.); ersiliaalexa@usvt.ro (E.A.)

**Keywords:** composite wheat flour with chestnut and rosehip, dough performance, bioactive compounds and antioxidant activity, functional bread, nutritional quality

## Abstract

Enriching bread with functional ingredients is a promising strategy to enhance the nutritional and bioactive profile of widely consumed foods. This study evaluated partial substitution of wheat flour (WF) with chestnut flour (CF) and rosehip powder (RP) on bread nutritional quality, functionality, and rheology. Five bread formulations were developed by replacing WF with CF at 0%, 5%, 10%, 15%, and 20%. Four other formulations were prepared by replacing WF in the 15% CF sample with RP at 0.5%, 1%, 2%, and 3%. Proximate composition, total phenolic content (TPC), total flavonoid content (TFC), antioxidant activity (DPPH and FRAP), and key physical characteristics were assessed, alongside the retention rates of functional attributes after baking. Rheological behavior of composite flours was analyzed using the MIXOLAB system to evaluate dough performance. Results showed that moderate WF substitution with CF (5–15%) increased dietary fiber and antioxidant activity while maintaining acceptable dough rheology and bread quality. At 20% CF substitution, TPC, TFC, FRAP, and DPPH increased 1.62-, 1.63-, 2.93-, and 3.03-fold versus control, with 59–66% retention. Addition of RP up to 3% to the 15% CF-substituted sample further enhanced bioactive properties, with TPC, TFC, FRAP, and DPPH reaching 2.13-, 2.03-, 4.49-, and 3.99-fold vs. BCF15, while retaining 61–67% of their functionality. Further inclusion of RP up to 2% in the 15% CF formulation maintains acceptable dough and bread performance, while 3% RP maximizes phytochemical enrichment but slightly affects technological properties. The combination of 15% CF and 2% RP provided a balanced enhancement in bioactive content and technological performance, offering a practical approach for producing functional bread with improved nutritional and technological attributes.

## 1. Introduction

The global shift toward healthier diets has driven the development of functional foods, including commonly consumed products such as bread. Growing interest in enhanced nutritional quality, improved sensory characteristics, and added health benefits has led to the incorporation of alternative ingredients into wheat-based formulations. In this context, alternative flours and bioactive powders are being examined as effective fortifying agents in bakery applications. Chestnut flour, derived from *Castanea sativa*, has a long culinary tradition. It is rich in complex carbohydrates and dietary fiber (4–10%), and provides essential minerals such as potassium and magnesium, B-group vitamins, and a low fat content (2–6%) [1,2]. Its natural sweetness and nutty flavor contribute positively to the sensory profile of baked products. However, the absence of gluten in chestnut flour reduces dough elasticity and gas retention, weakening structure and presenting challenges in rheological performance when combined with wheat flour. Several studies have examined the effects of chestnut powder on bread [3,4,5,6,7], as well as on muffins [8], wafer sheets [9], cookies [10,11,12], biscuits [13], and pasta [14]. Rheological studies showed that chestnut flour influences water absorption, dough stability, and extensibility [15,16]. Chestnut flour has been shown to improve the physico-chemical and functional properties of bread both immediately after baking and during storage [7]. Incorporating 10% chestnut flour has been reported to reduce bread volume by more than 50%, often resulting in denser and firmer crumbs [3,4]. However, Marciniak-Lukasiak et al. [5] found that the same substitution level can increase the bread volume. To compensate for the reduced viscoelasticity caused by the absence of gluten, hydrocolloids are commonly used as dough improvers [4]. By supplementing wheat flour with 20% and 50% chestnut flour, bread volatile profile, flavor complexity, and nutritional properties were improved; however, at 50% substitution, bread exhibited lower volume, a harder crumb, and darker color as a result of higher fiber and sugar content [17]. The gluten-free nature of chestnut flour makes it suitable for consumers with gluten-related disorders, but its use in gluten-free formulations requires careful adjustment to prevent negative effects on texture and sensory quality [4,16].

Studies indicate that incorporating chestnut flour and rosehip powder into bread and pastry products enhances their functional properties by increasing bioactive compounds including total phenolics and flavonoids. Chestnut flour is known for its high content of phenolic compounds and antioxidant potential [10,18,19]. Its addition to bread contributes to the improvement of functional quality [17]. Choi et al. [8] reported that higher levels of chestnut powder in rice muffins led to a significant rise in total polyphenol and flavonoid content, as well as antioxidant activity, confirming its beneficial effects in pastries.

Building on the exploration of chestnut flour as a functional ingredient, recent attention has also focused on rosehip powder, derived from various *Rosa* species. Similar to chestnut flour, rosehip powder offers valuable nutritional and technological benefits in bakery products, making it a promising additive to enhance both health-related properties and processing performance. Rosehip powder, derived from various *Rosa* species, has attracted interest as a functional ingredient due to its high content of ascorbic acid (vitamin C), carotenoids, polyphenols, and dietary fiber, which confer antioxidant and anti-inflammatory effects [20,21,22,23]. Its addition to dough influences gas formation and gluten structure, mainly through the oxidizing effect of ascorbic acid on sulfhydryl groups, as well as water absorption and dough stability [24,25].

Rosehip powder enhances both the nutritional and technological qualities of bakery products, improving characteristics in cookies [26] and sourdough bread, where it affects specific volume, crumb texture, and sensory acceptance [24,25,27,28]. Rheological studies reveal changes in farinographic properties and subsequent bread physico-chemical features with rosehip addition [24,27]. A 10% substitution with rosehip seed flour can double dietary fiber content compared to control bread [25]. Sensory effects include darker crumb color and modifications in bread volume, firmness, and chewiness, while lower concentrations may improve crust color, aroma, and flavor [24,25]. Additionally, rosehip powder delays staling by preserving crumb softness and elasticity, improves dough stability, and enhances sensory attributes such as crust color, aroma, and taste, although it may slightly reduce bread volume [27]. Its application also extends to other bakery products, including fiber-rich waffle cones, improving their nutritional profile [29]. Rosehip powder is rich in polyphenols and other high-value bioactive compounds, which correlate with its strong antioxidant activity [23,30]. Integration into products such as waffle cones successfully enhances polyphenol and carotenoid content improving functional attributes [29].

Ivanova et al. [31] investigated the effects of chestnut, rosehip, and pumpkin flours on wheat bread, showing that chestnut and rosehip addition significantly increased phenolic and flavonoid content, thereby enhancing antioxidant activity. Another study [32] reported that a balanced use of chestnut, rosehip, and pumpkin seed flours in wheat bread formulations helps maintain crumb softness and elasticity while delaying staling.

The technical rationale of our study is based on previous findings showing that moderate chestnut flour (CF) substitution levels (5–15%) enhance nutritional and fiber content while maintaining acceptable dough and bread characteristics [3], whereas higher levels (>20%) negatively affect dough rheology and baking performance [17]. Similarly, incorporation of rosehip powder at 1–3% improves antioxidant potential without substantially compromising dough properties, while higher inclusion levels can reduce dough stability and loaf quality due to its high acidity and fiber content [24,29].

Although the individual benefits of chestnut flour and rosehip powder in bread-making have been previously explored, their combined use remains insufficiently studied. To address this gap, the aim of this study was to investigate the effects of substituting wheat flour with chestnut flour (0–20%), alone or combined with rosehip powder (0.5–3% in the 15% chestnut flour-substituted sample), on dough performance, including rheological properties, nutritional and bioactive compound content, antioxidant profile, and key physical characteristics of bread. The practical purpose was to provide insights for the development of nutritionally enriched breads with improved functional properties, with a focus on the retention of these attributes after baking.

## 2. Materials and Methods

### 2.1. Chemicals and Reagents

All chemicals and reagents used in this study were of analytical grade. Standards such as gallic acid, quercetin, Trolox (6-hydroxy-2,5,7,8-tetramethylchroman-2-carboxylic acid), and ferrous sulfate heptahydrate (FeSO_4_·7H_2_O), along with reagents including 1,1-diphenyl-2-picrylhydrazyl (DPPH), 2,4,6-tris(2-pyridyl)-s-triazine (TPTZ), anhydrous sodium acetate, glacial acetic acid, hydrochloric acid (0.1 M), aluminum nitrate nonahydrate, and sodium nitrite were purchased from Sigma-Aldrich (Taufkirchen, Germany). Ethanol (96%) was supplied by Chimreactiv (Bucharest, Romania), while Folin–Ciocalteu reagent, ferric chloride hexahydrate, and anhydrous sodium carbonate were obtained from Merck (Darmstadt, Germany).

### 2.2. Bread Ingredients and Manufacturing Process

The ingredients used in bread production included wheat flour type 650 (WF) with a moisture content of 12.74%, marketed as Baneasa, purchased from METRO Cash and Carry in Arad, Romania; chestnut flour (CF) from Targroch and rosehip powder (RP) from BioPlanet, both obtained from the VitaMix natural goods store in Arad, Romania. Chestnut flour (CF) and rosehip powder (RP) were passed through an 80-mesh **sieve** to achieve uniform particle size. Additional ingredients included salt sourced from S.C OLYMPIA SRL, Romania and fresh yeast Bravo produced by ROMPAK SRL (Pascani, Romania), both acquired from a local supermarket.

**Figure 1:**
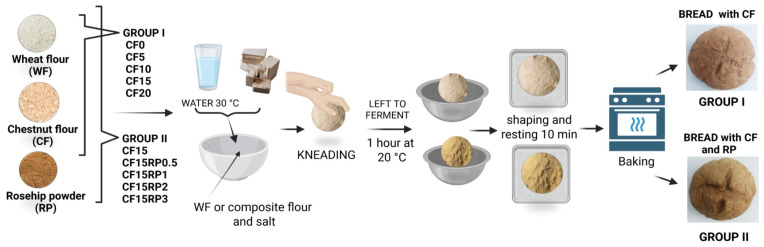
Schematic representation of the technological process used to obtain fortified bread from Group I and Group II composite flours (Figure created with BioRender.com). Group I (CF0, CF5, CF10, CF15, and CF20): wheat flour with 0%, 5%, 10%, 15%, and 20% chestnut flour. Group II (CF15RP0.5, CF15RP1, CF15RP2, and CF15RP3): wheat flour with 15% chestnut flour plus 0.5%, 1%, 2%, and 3% rosehip powder.

Two groups of composite flours were prepared by partially substituting wheat flour (WF). In the first group, WF was replaced with chestnut flour (CF) at 0% (control), 5%, 10%, 15%, and 20%, yielding CF0–CF20 blends. In the second group, rosehip powder (RP) was incorporated into the 15% CF blend (CF15) at 0.5%, 1%, 2%, and 3%, yielding CF15RP0.5–CF15RP3 blends. Composite flour samples for chemical analysis were placed in polypropylene bags and stored at −20 °C until further investigations. Bread formulations derived from the obtained blends were labeled BCF0–BCF20 for Group I and BCF15–BCF15RP3 for Group II, while the corresponding doughs were designated DCF0–DCF20 and DCF15–DCF15RP3, respectively. Bread formulations were prepared following a slightly modified single-phase method as described by Raczyk et al. [33]. The base recipe corresponded to a conventional formula of 1 kg wheat flour, 600 mL water, 25 g fresh yeast, and 20 g salt. Each experimental batch was standardized to 200 g total flour, with ingredient ratios adjusted accordingly, as follows: 200 g flour (wheat flour or composite), 5 g fresh yeast, 4 g salt, and 120 mL water (60% hydration, preheated to 30 ± 1 °C). A constant hydration level, validated in preliminary trials, was maintained across all formulations to enable direct comparison under uniform experimental conditions. Fresh yeast was dissolved in warm water (30 °C) and gradually incorporated into the flour and salt blend. Mixing was performed by manual kneading for 6–8 min until the dough became smooth and elastic. The kneaded dough was transferred to a covered bowl and fermented at ambient temperature (≈20 °C) for 1 h, with one fold performed at the 30 min mark to redistribute gases. After fermentation, the dough was divided into three equal portions, each hand-rounded into smooth, uniform balls. Portions were covered and rested at ambient temperature for 10 min to relax the gluten network, then shaped and placed on parchment-lined baking trays. Baking was performed in a professional convection oven (FM RXB 610 V7; 13,650 W, 400 V, 50/60 Hz; FM Industrial S.A., Lucena, Córdoba, Spain). The oven was preheated to 250 °C for 20 min with a stainless-steel bowl of water on the upper shelf for steam generation. After 30 min of preheating and initial steaming, dough pieces were baked at 250 °C for 12 min. The water bowl was then removed, and baking continued at 200 °C for 20 min. Figure 1 illustrates the schematic process for producing fortified bread from Group I and Group II composite flours.

Breads were cooled on wire racks at ambient temperature for 12 h before further handling. All formulations were prepared in triplicate (n = 3), with three samples produced per replicate. For each batch and formulation, samples were collected both from the dough (prior to baking) and from the corresponding bread. Samples were placed in polypropylene bags and stored at −20 °C until chemical analysis. Moisture content and physical characteristics of the bread were assessed immediately after the 12 h cooling period.

### 2.3. Proximate Composition and Energy Value Assessment of Wheat Flour, Chestnut Flour, Rosehip Powder, and Enriched Bread Samples

The proximate composition and energy content of WF, CF, RP, and enriched breads were determined using standardized methods established by the Association of Official Analytical Chemists (AOAC) [34]. Moisture content was measured according to AOAC Official Method 925.10 by drying samples in an electric oven at 105 °C until a constant weight was reached. Crude fat content was assessed using AOAC Official Method 922.06, and protein content was evaluated by AOAC Official Method 920.87. Ash content was quantified by incineration in a muffle furnace at 550 °C. Fiber content analysis was performed based on AOAC Official Method 991.43. Carbohydrate content was calculated by subtracting the combined percentages of protein, ash, fat, and moisture from 100%.

Energy value (kcal per 100 g) was calculated using Atwater conversion factors, which assign 4 kcal/g to carbohydrates and protein, and 9 kcal/g to fat [35], following Equation (1):

Energy value (kcal/100 g) = 4 × Carbohydrates (%) + 9 × Lipids (%) + 4 × Proteins (%) (1)

### 2.4. Rheological Determination of Composite Flours Using MIXOLAB System

Rheological properties of the enriched flours were evaluated using the MIXOLAB device (Chopin+, Chopin Technologies, Paris, France) following the ICC Standard Method [36] and Chopin+ protocol [37]. For each analysis, 50 g of flour was placed in the MIXOLAB bowl, and water was automatically added to reach the target dough consistency. The test involved mixing at 80 rpm, with temperature plateaus at 30 °C for 8 min and 90 °C for 7 min, followed by a 5 min phase at 50 °C, with heating at 4 °C/min. Key parameters recorded included water absorption, dough development time, dough stability, maximum torque generated in the mixing phase (C1), protein weakening (C2), starch gelatinization (C3), torque reduction (C4), and final torque after cooling (C5) [36].

### 2.5. Phytochemical Content and Antioxidant Activity of Wheat Flour, Chestnut Flour, Rosehip Powder, Dough, and Enriched Bread

#### 2.5.1. Preparation of Alcoholic Extracts from Samples

Ethanol-based extracts were prepared to assess total phenolic content, total flavonoid content, and antioxidant activity, following an adapted method from Litwinek et al. [38]. Approximately 1.0 g of sample (WF, CF, RP, dough, or bread) was mixed with 10 mL of 70% ethanol (*v*/*v*) and stirred at room temperature for 2 h using an IDL magnetic stirrer (IDL GmbH & Co. KG, Nidderau, Germany). After centrifugation at 10,000 rpm (rotor radius 9.5 cm) for 10 min (Hettich EBA 21, Andreas Hettich GmbH & Co. KG, Tuttlingen, Germany), the residue was re-extracted with 70% ethanol for 1 h under continuous stirring and centrifuged again under the same conditions. Supernatants from both extractions were combined and stored at −20 °C in the dark until analysis. Samples were processed in triplicate, with each replicate used independently in subsequent assays.

#### 2.5.2. Determination of Total Flavonoid Content

Total flavonoid content (TFC) was assessed using a modified method adapted from Al-Farsi et al. [39]. In brief, 3.0 mL of the ethanol extract was combined with 4.5 mL distilled water and 1.0 mL of 0.3% sodium nitrite (NaNO_2_) solution. The mixture was incubated at 20 °C for 6 min, then 1.0 mL of 10% aluminum nitrate [Al(NO_3_)_3_] solution was added and incubated for an additional 6 min. Next, 10 mL of 4% (*w*/*w*) sodium hydroxide (NaOH) was added, then the volume adjusted to 25 mL using 70% ethanol (*v*/*v*). After 15 min, absorbance was recorded at 510 nm against a 70% ethanol blank. Quantification was based on a quercetin calibration curve (0.5–50 µg/mL), with results expressed as mg quercetin equivalents (QE) per 100 g dry weight (d.w.).

#### 2.5.3. Determination of Total Phenolic Content

Total phenolic content (TPC) quantification was carried out using the Folin–Ciocalteu assay following the methodologies described by Blanch et al. [40]. The alcoholic extracts were used undiluted for TPC analysis, except for the RP sample, which was diluted with distilled water at a 1:10 (*v*/*v*) ratio prior to analysis. Then, 0.5 mL of the resulting extract was mixed with 2.5 mL of Folin–Ciocalteu reagent, previously diluted 1:10 (*v*/*v*) with distilled water. Following this, 2 mL of a 7.5% Na_2_CO_3_ solution was added. The obtained mixture was incubated for 30 min at 50 °C in a Memmert GmbH INB500 thermostat (Schwabach, Germany), and the absorbance was then measured at 750 nm using a Specord 205 UV–Vis spectrophotometer (Analytik Jena AG, Jena, Germany) against a blank sample prepared under identical conditions. Total phenolic content (TPC) was quantified using a gallic acid calibration curve (0.1–1.0 μM GAE/mL) and expressed as mg gallic acid equivalents (GAE) per 100 g d.w.

#### 2.5.4. Evaluation of Antioxidant Activity via 1,1-Diphenyl-2-Picrylhydrazyl (DPPH) Assay

The free radical scavenging ability of the samples was measured using the DPPH assay [41], a widely applied method for assessing antioxidant activity based on the capacity to quench free radicals. The assay was carried out using a 0.1 mM DPPH solution prepared in 70% ethanol. The extracts obtained from WF, CF, dough, and bread samples were further diluted with 70% (*v*/*v*) ethanol at a ratio of 1:5 (*v*/*v*), while the extract derived from RP was diluted at a ratio of 1:100 (*v*/*v*). A 1.0 mL aliquot of the diluted extract was added to 2.5 mL of a 0.1 mM DPPH solution prepared in 70% ethanol. The mixture was thoroughly stirred using an IDL hot plate stirrer (IDL GmbH & Co. KG, Nidderau, Germany) and then incubated in the dark at 20 °C for 30 min. Absorbance was measured at 517 nm, using 70% ethanol as the blank. A control sample was prepared under the same conditions by mixing 1 mL of 70% ethanol with 2.5 mL of the DPPH solution. The antioxidant activity of the samples, expressed as DPPH radical scavenging, was calculated according to Equation (2):

(2) $$\mathrm{DPPHRadical}\;\mathrm{Scavenging}\;\mathrm{Activity}\;(\%)=\frac{A_{\mathrm{control}}-A_{\mathrm{sample}}}{A_{\mathrm{control}}}\times 100$$

where A_control_ represents the absorbance of the control sample, and A_sample_ refers to the absorbance measured for the test sample. The DPPH scavenging activity (%) was plotted against Trolox concentrations (µg/mL) to establish a calibration curve using Trolox standard solutions ranging from 1.0 to 25 µg/mL [42], from which Trolox equivalent (TE) concentrations were calculated. Antioxidant activity was then determined by factoring in the molar mass of Trolox and the concentration of the sample solution (g/mL), with results expressed as µmol TE/g d.w.

#### 2.5.5. Evaluation of Antioxidant Activity via Ferric-Reducing Antioxidant Power (FRAP) Assay

The antioxidant capacity of the test samples including WF, CF, RP, dough, and the resulting bread was measured using the ferric-reducing antioxidant power (FRAP) assay. This method relies on the ability of antioxidant compounds in ethanol extracts to donate electrons, reducing ferric ions (Fe^3+^) in a colorless ferric tripyridyltriazine complex to ferrous ions (Fe^2+^) under acidic conditions. This reaction forms a blue Fe^2+^ tripyridyltriazine complex which exhibits maximum absorbance at 593 nm [43]. The FRAP reagent was made by mixing 100 mL of acetate buffer at pH 3.6 with 10 mL of a 10 mM TPTZ solution prepared in 40 mM hydrochloric acid, along with 10 mL of a 20 mM ferric chloride hexahydrate (FeCl_3_·6H_2_O) solution. The alcoholic extracts obtained from WF and from dough and bread samples enriched with CF were used undiluted for FRAP analysis. In contrast, extracts from CF and from dough and bread samples containing RP were diluted with distilled water at a 1:5 (*v*/*v*) ratio prior to analysis. The extract obtained directly from RP was further diluted at a ratio of 1:100 (*v*/*v*). Subsequently, 0.5 mL of each extract was combined with 2.5 mL of the FRAP working solution and incubated at 37 °C for 30 min. Absorbance was then measured at 593 nm against a blank prepared under identical conditions. FRAP values were determined based on a calibration curve prepared using standard solutions of ferrous sulfate heptahydrate (FeSO_4_·7H_2_O), in the concentration range of 0.05 to 0.5 µM. The results were expressed as µM Fe^2+^/g d.w.

### 2.6. Physical Characteristics Assessment

The bread samples were evaluated for several key physical parameters, such as porosity, crumb elasticity, and height-to-diameter (H/D) ratio, according to SR 91:2007 [44]. All measurements were performed in triplicate for each bread sample.

Porosity was expressed as the percentage of pore volume in 100 g of crumb and calculated according to Equation (3):

(3) $$\mathrm{Porosity}\;(\%,\mathrm{vol}.)=\frac{V-\frac{m}{\rho }}{V}\times 100$$

where V is the volume of the crumb cylinder (cm^3^), m is the mass of the crumb cylinder (g), and *ρ* is the density of the compact crumb (g/cm^3^). The *ρ* value specified by SR 91:2007 [44] is 1.26 g/cm^3^ for semi-white wheat bread. This method is based on measuring the density of the pore-free core.

Crumb elasticity, calculated according to Equation (4), was determined by compressing a cylindrical crumb piece (60 mm in height) for 1 min, followed by measuring its recovery.

(4) $$\mathrm{Elasticity}\;(\%)=\frac{B}{A}\times 100$$

where A represents the height of the core cylinder before pressing, and B represents its height after pressing, once it has returned.

The height-to-diameter ratio (H/D) was determined by measuring the maximum height of the bread (H, cm) and the arithmetic mean of two perpendicular diameters (D, cm). It was calculated using the formula presented in Equation (5):

(5) $$\mathrm{Height}\;/\;\mathrm{Diameter}\;r\;\mathrm{ratio}=\frac{H}{D}$$

### 2.7. Statistical Analysis

Experimental data were obtained from three independent trials, each with three analytical replicates. Results are reported as mean ± standard deviation (SD) across these trials. Statistical differences among formulations were evaluated by one-way ANOVA, followed by Tukey’s post hoc test for multiple comparisons. Levene’s test was used to verify homogeneity of variances. The assumptions of homogeneity, normality (or approximate normality) of residuals, and independence were confirmed. Differences were considered statistically significant at *p* < 0.05.

## 3. Results and Discussion

### 3.1. Proximate Composition of Wheat Flour, Chestnut Flour, Rosehip Powder, and Enriched Bread Formulations

The proximate composition of wheat flour (WF), chestnut flour (CF) and rosehip powder (RP) is shown in Table 1.

Significant differences (*p* < 0.05) in proximate composition were observed among WF, CF, and RP. RP exhibited the lowest moisture content (7.258%) compared to WF (12.74%) and CF (11.52%). CF showed a higher lipid content (3.91%) relative to WF (1.56%), as well as increased dietary fiber (7.25% vs. 3.41%) and ash content (3.01% vs. 0.64%), but lower protein levels (7.79% vs. 12.89%). These elevated levels of lipids, fiber, and minerals suggest that CF is a valuable raw material with enhanced nutritional potential compared to WF, as supported by studies reporting similar compositional values [33,45]. Table 1 shows that RP was characterized by a lipid content of 4.87%, protein content of 6.49%, ash content of 4.49%, and a high dietary fiber level of 56.09%, with an energy value of 377.35 kcal/100 g, in close agreement with the results reported in previous studies [24,29].

**Figure 2:**
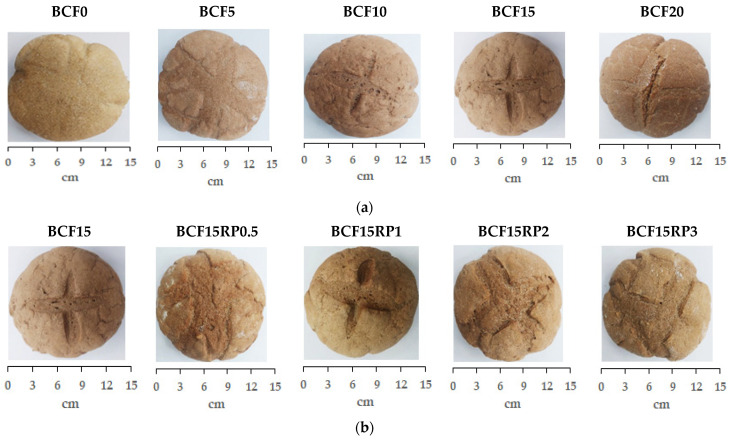
Bread samples from wheat flour with chestnut flour (**a**) and with chestnut flour plus rosehip powder (**b**). Group I (BCF0, BCF5, BCF10, BCF15, and BCF20): bread from wheat flour with 0%, 5%, 10%, 15%, and 20% chestnut flour. Group II (BCF15RP0.5, BCF15RP1, BCF15RP2, and BCF15RP3): bread from wheat flour with 15% chestnut flour plus 0.5%, 1%, 2%, and 3% rosehip powder.

Figure 2 illustrates the fortified bread obtained from composite flours belonging to Group I and Group II, while Table 2 presents their proximate composition.

The developed bread formulations, enriched with CF and with 15% CF combined with RP, demonstrated an improved nutritional profile compared to the corresponding controls (BCF0 for CF-fortified samples and BCF15 for the 15% CF sample supplemented with RP). The improvements in nutritional profile gradually increased with the incremental substitution levels of WF by CF, and by the combined addition of CF and RP. The results indicated that the inclusion of CF and RP significantly affected (*p* < 0.05) the proximate composition of the bread formulations.

The moisture content of the bread samples showed a slight decreasing trend with increasing CF or RP supplementation. In Group I, values ranged from 37.72% in BCF0 to 37.59% in BCF20, and in Group II, from 37.62% in BCF15 to 37.59% in BCF15RP3 (Table 2). Statistical analysis indicated no significant differences among samples in either group (*p* > 0.05). Despite this, the observed moisture values were slightly higher than those reported by Raczyk et al. [33], who found 35.61% in bread with 15% wheat flour substitution by CF. The slight decrease in moisture content may be attributed to the increased dietary fiber from CF and RP, which can reduce the water-holding capacity of the dough [46].

Protein content decreased significantly with increasing substitution of WF by CF in Group I, from 9.55% in BCF0 to 8.79% in BCF20 (*p* < 0.05). In contrast, in 15% CF-substituted samples with added RP (Group II), protein content ranged from 8.96% in BCF15RP0.5 to 8.84% in BCF15RP3, with no significant differences among samples (*p* > 0.05), indicating that RP addition did not affect protein level. The highest protein content was observed in the control sample BCF0. These findings align with Demirkesen et al. [4] and Vartolomei and Turtoi [24], who reported that CF and RP are low-protein ingredients, contributing to the reduced protein content in bread compared to WF.

In terms of lipid content, a significant increase was observed in CF-enriched samples (Group I), from 1.15% in the control (BCF0) to 1.50% in BCF20 (*p* < 0.05). In contrast, in 15% CF-enriched samples with added RP (Group II), lipid content ranged from 1.41% (BCF15) to 1.49% (BCF15RP3), with no significant differences among samples (*p* > 0.05), indicating that RP addition did not affect lipid levels. Overall, the lipid levels in all samples were lower than those reported by Raczyk et al. [33], who documented values of 1.90–2.63% in similar bread products.

The developed formulations showed a significant increase in dietary fiber content, ranging from 2.53% (BCF0) to 3.09% (BCF20) in CF-enriched bread), and from 2.95% (BCF15) to 4.10% (BCF15RP3) in 15% CF-enriched bread with added RP (*p* < 0.05). These findings, consistent with Dall’Asta et al. [17] and Vartolomei and Turtoi [24], confirm the high fiber content of CF and RP. Increased dietary fiber enhances the nutritional profile of bread, though it may also influence dough rheology and final product quality [47].

Ash content showed a significant increasing trend in CF-enriched bread (Group I), ranging from 0.48% in BCF0 to 0.83% in BCF20, while in 15% CF-enriched bread with added RP (Group II), values ranged from 0.74% in BCF15 to 0.82% in BCF15RP3, with no significant differences among samples (*p* > 0.05) (Table 2). These results reflect the mineral content of the bread and are consistent with previous studies. For instance, Vartolomei et al. [48] found that supplementation of wheat bread with 5% CF and 10% RP increased mineral content, and Zlateva et al. [49] similarly confirmed that enriching wheat bread with CF and RP is an effective approach to improving dietary mineral intake.

Statistical analysis indicated no significant differences in carbohydrate content among the bread samples (*p* > 0.05). In CF-fortified bread (Group I), values ranged from 51.10% in the control (BCF0) to 51.29% in BCF20, while in 15% CF-enriched bread with added RP (Group II), values ranged from 51.24% in BCF15RP0.5 to 51.26% in BCF15RP3 (Table 2). The carbohydrate levels reported in this study closely match the values documented by Borșa et al. [29], when adjusted to dry weight equivalents of the supplemented bread samples.

Increasing the supplementation level, both in CF-enriched samples and in RP-containing samples (CF15RP0.5–CF15RP3), led to only slight increases in energy value, as shown in Table 2. Statistical analysis revealed that in Group I (BCF0–BCF20), energy values increased significantly with higher CF content, whereas in Group II (BCF15 with 0.5–3% added RP), no significant differences were observed among samples (*p* > 0.05).

These results indicate that incorporation of CF and RP into bread formulations has a limited effect on caloric content while improving the nutritional profile, particularly in lipid, fiber, and ash content.

These results indicate that increasing CF level (Group I) significantly affects certain nutritional components, such as fat, fiber, and ash, while caloric content changes remain slight. In contrast, adding RP to 15% CF-substituted bread (Group II) does not significantly affect fat, protein, ash, or carbohydrate content, but it does significantly increase dietary fiber. Overall, increasing CF and adding RP can improve the nutritional profile of bread without markedly affecting its caloric content.

### 3.2. Rheological Properties of Composite Flours

**Figure 3:**
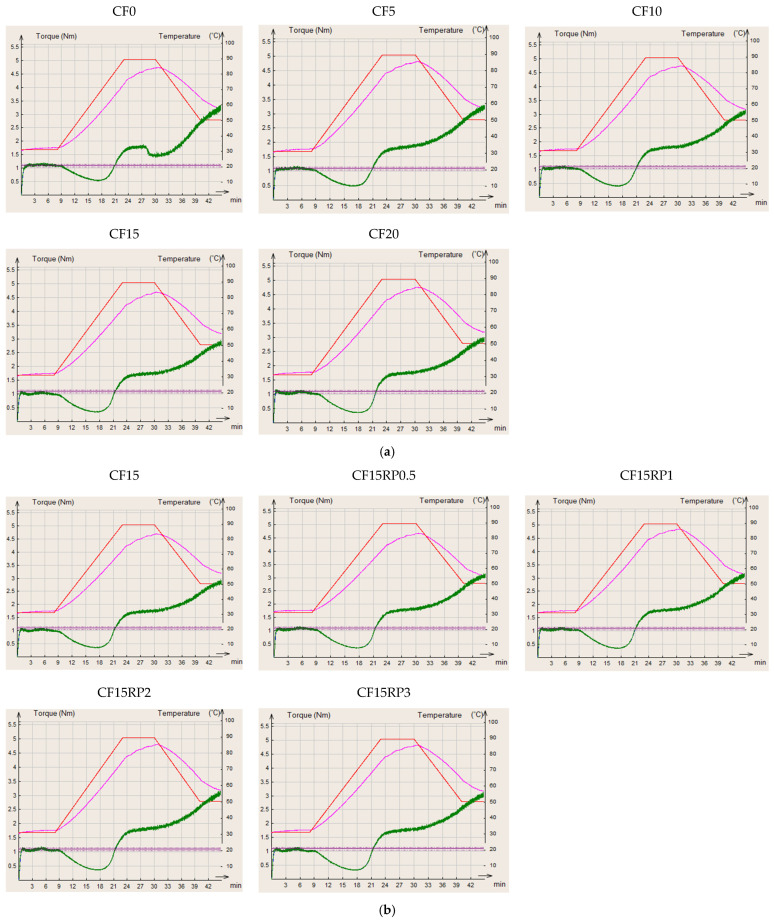
MIXOLAB rheological profiles of wheat flour with chestnut flour (**a**) and from wheat flour with chestnut flour plus rosehip powder (**b**). Group I (CF0, CF5, CF10, CF15, and CF20): wheat flour with 0%, 5%, 10%, 15%, and 20% chestnut flour. Group II (CF15RP0.5, CF15RP1, CF15RP2, and CF15RP3): wheat flour with 15% chestnut flour plus 0.5%, 1%, 2%, and 3% rosehip powder. Red line: MIXOLAB temperature (°C); pink line: dough temperature (°C); green line: MIXOLAB curve.

The rheological behavior of the composite flours, assessed using the MIXOLAB (CHOPIN Technologies), allowed evaluation of key dough properties including water absorption, dough stability, protein weakening, starch gelatinization, and enzymatic activity during heating and cooling. These measurements provide a comprehensive profile of the dough’s performance under simulated baking conditions, reflecting the quality of both protein and starch [37]. The Mixolab curves for all formulations are shown in Figure 3.

**Figure 4:**
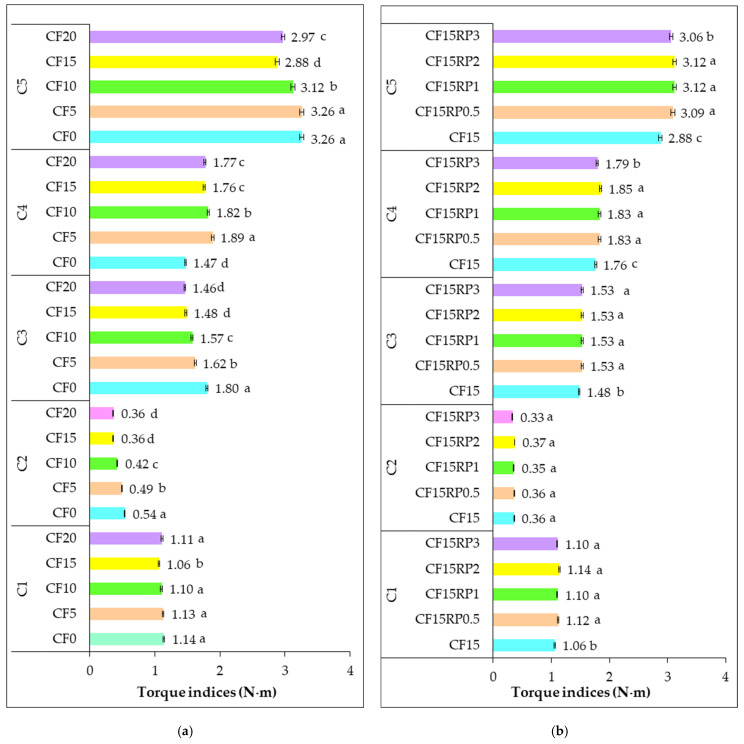
Torque indices (N·m) for wheat flour with chestnut flour (**a**), and with chestnut flour plus rosehip powder (**b**). C1: maximum torque during mixing; C2: torque reflecting protein weakening by mechanical stress and increasing temperature; C3: torque reflecting rate of starch gelatinization; C4: minimum torque during heating; C5: torque after cooling to 50 °C. Group I (CF0, CF5, CF10, CF15, and CF20): wheat flour with 0%, 5%, 10%, 15%, and 20% chestnut flour. Group II (CF15RP0.5, CF15RP1, CF15RP2, and CF15RP3): wheat flour with 15% chestnut flour plus 0.5%, 1%, 2%, and 3% rosehip powder. Data are expressed as mean ± standard deviation of three independent analyses. Different letters above columns indicate significant differences within the same parameter (one-way ANOVA, *p* < 0.05), while those with the same letter are not significantly different (*p* > 0.05).

The analysis of torque indices (C1 to C5), shown in Figure 4, illustrates the effects of partially replacing WF with CF at levels from 0% to 20%, as well as adding 0.5%, 1%, 2%, and 3% RP to the 15% CF formulation, on dough rheological properties.

Analysis of the results obtained for the pairs (C1–C5) by Mixolab in Figure 4 reveals contrasting effects of incorporating CF alone (Figure 4a) and in combination with RP (Figure 4b) on the rheological properties of wheat dough.

Particular attention was given to torque C1, which is mainly influenced by the amount of water absorbed by the flour blend. Initially, increasing the proportion of chestnut flour (0–20%) does not significantly affect torque C1 (maximum torque during kneading), as it remains broadly stable regardless of the percentage substitution (Figure 4a). In fact, the values were 1.14, 1.13, 1.10, 1.06, and 1.11 N·m for CF0, CF5, CF10, CF15, and CF20, respectively. This reflects a relatively preserved initial gluten structuring capacity. This finding is consistent with the observations of Collar et al. [50] who noted that the incorporation of alternative flours does not always affect the initial formation of the gluten network (kneading phase), but has a stronger effect during heating. The same observation was made when rosehip powder was added to sample CF15 (Figure 4b). Couple C1 remained stable (between 1.06 and 1.14 N·m), confirming that the residual gluten retains its initial structuring function.

On the other hand, torque C2 (an indicator of protein weakening due to mechanical and thermal stress) decreased significantly with increasing CF level (Figure 4a). In fact, C2 decreased from 0.54 N·m for CF0 to 0.36 for CF20. This reflects an increased weakening of the protein network under the combined effect of mechanical shearing and temperature elevation [37]. This reduction is attributable to the dilution of gluten and the interference of non-gluten components (fibers, simple sugars, and polyphenols) in CF, which corroborates the results of [16] showing that chestnut flour proteins do not compensate for the structuring function of gluten. Regarding the substitution of CF15 by RP (Figure 4b), C2 does not vary significantly with the addition of RP (0.36, 0.36, 0.35, 0.37, and 0.33 N·m for CF15RP0.5, CF15RP1, CF15RP2, and CF15RP3, respectively). This suggests that the bioactive components of RP do not enhance protein stability, with the dilution of gluten by chestnut flour remaining the dominant factor. This behavior is consistent with the conclusions of Demirkesen et al. [16], according to which gluten-free flours do not improve the resilience of the protein network but rather modify interactions with water.

About starch, pairs C3 (starch gelatinization) and C4 (starch gel thermal stability) reveal a gradual decrease in gelatinization intensity and starch gel thermal stability as the proportion of CF increases (Figure 4a). On the other hand, the addition of RP leads to a notable improvement in starch-related indices. Indeed, C3 increases slightly (from 1.48 to 1.53 between CF15 and CF15RP3), reflecting better water retention and more efficient structuring of the starch network. This observation is consistent with Mariotti et al. [51], who showed that fiber-rich ingredients alter water availability and promote the formation of more cohesive starch gels. Similarly, the C4 couple improves with increasing RP, reaching a maximum at 2% (1.85 N·m), suggesting a probable interaction between RP phenolic compounds and starch, reinforcing the thermal resistance of the dough.

Finally, the C5 couple (starch retrogradation after cooling) gradually decreases (3.26, 3.26, 3.12, 2.88, and 2.97 for CF0, CF5, CF10, CF15, and CF20, respectively) with increasing CF content (Figure 4a), indicating lower starch retrogradation after cooling. This effect can be considered beneficial in terms of the preservation of finished products, as it could limit the staling of bread or bakery products, which is consistent with the observations of Gómez et al. [52] on the incorporation of fiber-rich flours. About the addition of RP to CF15 (Figure 4b), the samples containing RP obtained a higher value than CF15. This increase reflects a more pronounced retrogradation, probably induced by the ability of soluble fibers and polyphenols to influence starch recrystallization. Although this retrogradation may increase crumb firmness, it could also accelerate staling, in contrast to the expected effects of other high-fiber flours [52]. The balance between functional benefits (firmer texture, and nutritional enrichment with fiber and antioxidants) and technological constraints (potential staling) must therefore be taken into account.

Thus, the incorporation of chestnut flour alone tends to weaken the protein structure and reduce starch gelatinization and retrogradation, which can improve shelf life but adversely affect texture and volume. Conversely, the addition of rosehip powder (1–2%) would improve gelatinization and thermal stability, while increasing post-cooling retrogradation. A technological compromise can therefore be sought as follows: moderate levels of CF (5–10%) enrich the dough with fiber and nutrients without excessively altering its properties, while a combination of 15% CF and RP (1–2%) strengthens the starch structure, at the cost of increased retrogradation. These results confirm the benefits of diversifying flours and functional powders, but highlight the importance of adjusting proportions to balance nutritional value, sensory quality, and technological stability.

**Figure 5:**
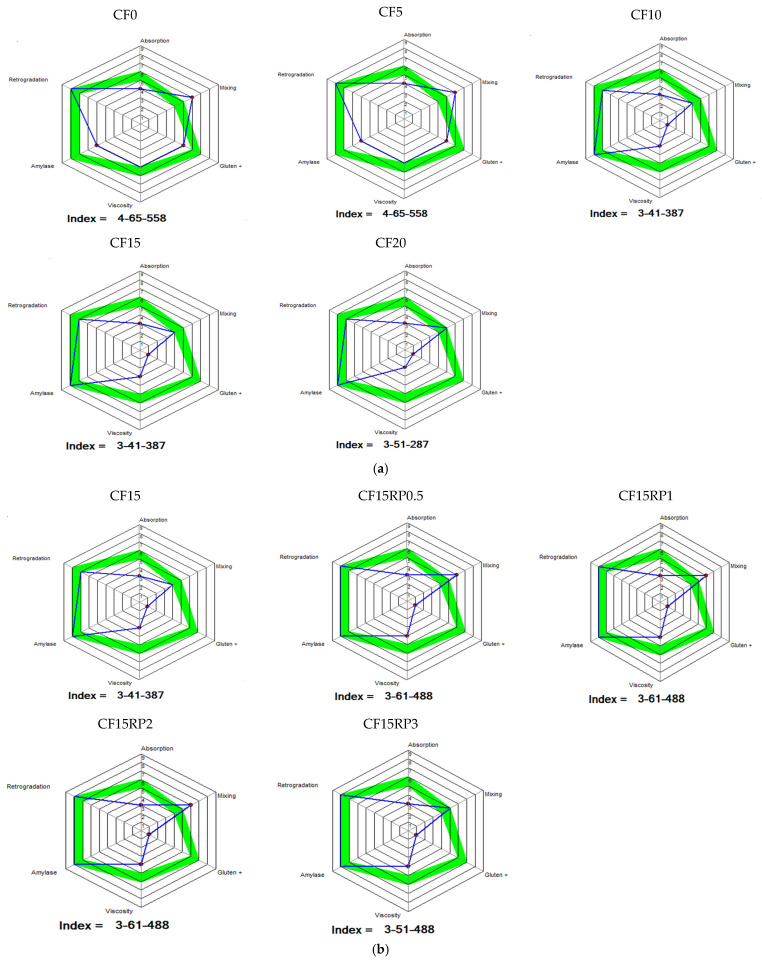
MIXOLAB Profiler Index of dough from wheat flour with chestnut flour (**a**) and from wheat flour with chestnut flour plus rosehip powder (**b**). Group I (CF0, CF5, CF10, CF15, and CF20): wheat flour with 0%, 5%, 10%, 15%, and 20% chestnut flour. Group II (CF15RP0.5, CF15RP1, CF15RP2, and CF15RP3): wheat flour with 15% chestnut flour plus 0.5%, 1%, 2%, and 3% rosehip powder. The blue line represents the profile of dough prepared from composite flours in Group I and Group II, while the green line shows the reference profile provided by the Mixolab software, used for comparison in evaluating the rheological characteristics of dough in bread-making.

The MIXOLAB Profiler Index offers a comprehensive evaluation of dough quality by summarizing the behavior of flour and water systems under simultaneous mechanical and thermal stress. As illustrated in Figure 5, this index uses a radar plot to compare the actual dough performance (blue line) with a bread-making reference profile (green line), provided by the Mixolab software. It analyzes six key parameters: water absorption, mixing, gluten network strength, amylase activity, protein weakening, and starch retrogradation, providing a numerical value that reflects the overall quality impact of WF substitution with CF and RP).

Retrogradation, a key factor influencing starch reassociation, staling, and shelf life, remained high in the control (CF0) and CF5. Increasing the substitution with CF led to a moderate decrease in retrogradation in CF10, CF15, and CF20, indicating a weakening of starch reassociation and a potential increase in susceptibility to staling [53,54]. The addition of RP to CF15 increased retrogradation compared with CF15, suggesting that bioactive compounds interact with starch and inhibit recrystallization, consistent with reports on polyphenol effects on amylose complexation [55]. At the highest RP concentration tested, retrogradation did not further increase, possibly due to saturation of interactions or interference with starch gel structure, a phenomenon supported by the literature describing structural weakening at high polyphenol concentrations [56].

Amylase activity, as reflected by viscosity changes during the heating phase in the Mixolab Profiler, was lower in the control (CF0) and CF5, indicating reduced enzymatic activity possibly due to differences in flour composition and substrate accessibility. CF10–CF20 exhibited higher enzymatic activity, reflecting more efficient starch hydrolysis [57,58]. The addition of rosehip powder (RP) to the CF15 formulation (0.5–3% RP, CF15RP0.5–CF15RP3) did not reduce amylase activity, indicating that RP incorporation at these levels did not inhibit α-amylase. These observations are consistent with previous studies showing that phenolic compounds can inhibit α-amylase in a dose- and matrix-dependent manner [58,59,60], but in this case, dough composition and water distribution maintained enzyme functionality.

Water absorption influences dough rheology and baking properties by affecting protein and starch hydration, gluten network formation, and starch gelatinization. In the present samples, absorption was higher in CF0 and CF5, while CF10–CF20 and CF15 with RP showed slightly lower values, indicating a modest reduction in water-holding capacity at higher chestnut flour levels and with RP addition. This slight decrease may result from interactions between CF fibers and gluten or changes in starch hydration at higher substitution levels. Soluble fibers and carbohydrates in CF and RP maintained water retention, with absorption remaining constant at higher substitution levels. Dietary fiber and polyphenol-rich ingredients in CF and RP support water absorption due to their hydrophilic properties, maintaining stable values at higher substitution levels [56,61].

Mixing stability reflects dough resistance to mechanical shear, which is important for dough tolerance and baking quality. CF0 and CF5 showed relatively high mixing stability, indicating good dough resistance. CF10 and CF15 exhibited lower mixing stability, reflecting weaker gluten structure and reduced resistance to mechanical stress. CF20 showed a slight increase, indicating partial recovery of dough strength. The addition of rosehip powder (RP) to CF15 improved mixing stability at 0.5–2% RP, while 3% RP showed a moderate improvement. This enhancement is attributed to RP fibers and bioactive compounds interacting with the dough by absorbing water and stabilizing its structure. These findings are consistent with previous studies reporting that dietary fibers and polyphenol-rich additives enhance mixing tolerance and dough stability through water retention and gluten-plasticizing effects [61,62].

Gluten performance is essential for gas retention and dough structure. CF0 and CF5 showed moderate gluten strength. Increasing chestnut flour levels from CF10 to CF20 resulted in reduced gluten strength, indicating that higher CF substitution weakens the protein network. The addition of rosehip powder (RP) to CF15 did not improve gluten performance. All RP-containing samples (CF15RP0.5–CF15RP3) exhibited reduced gluten strength, due to organic acids and polyphenols interfering with gluten formation, which decreases elasticity and dough cohesion.

Viscosity reflects starch gelatinization and swelling during heating, which are critical for gas retention, crumb structure, and baking performance. CF0 and CF5 showed high viscosity, indicating well-developed starch gelatinization. Increasing the substitution of WF with CF led to a gradual decrease in viscosity, with CF10 and CF15 exhibiting lower viscosity and CF20 showing the most pronounced reduction compared to the control. This decrease can be attributed to the lower starch content at higher CF levels, which reduces the amount of starch available for gelatinization, and to altered water-binding properties of the dough, which limit starch swelling and gel formation. As a result, the dough has a weaker capacity to form a cohesive gel network, contributing to lower viscosity.

The addition of RP to CF15 led to a moderate increase in viscosity across all tested concentrations (CF15RP0.5–CF15RP3), indicating that RP components contribute to starch swelling and water retention in the dough. At lower RP levels (CF15RP0.5 and CF15RP1), viscosity was slightly higher than CF15, suggesting synergistic interactions between RP fiber and starch that enhance water binding and gel formation. Higher RP levels (CF15RP2 and CF15RP3) maintained similar viscosity values, indicating a stabilizing effect of RP on dough structure even at elevated levels. Overall, RP addition improved dough viscosity compared with CF15, highlighting its positive influence on dough properties.

Phenolic compounds have been reported to reduce pasting viscosity in a dose-dependent manner by forming hydrogen bonds with starch and limiting granule swelling [63,64]. Wu et al. [58] explained that polyphenols can form complexes with starch, disrupting inter-chain interactions, decreasing swelling, and reducing viscosity, with stronger effects at higher concentrations. In this study, however, the addition of RP in the range of 0.5–3% did not decrease dough viscosity. This can be explained by the relatively low RP content and the fiber matrix, which supports water retention and starch swelling, counteracting potential inhibitory effects of polyphenols. As a result, RP addition stabilized viscosity across all tested levels, highlighting a positive effect on dough properties.

**Figure 6:**
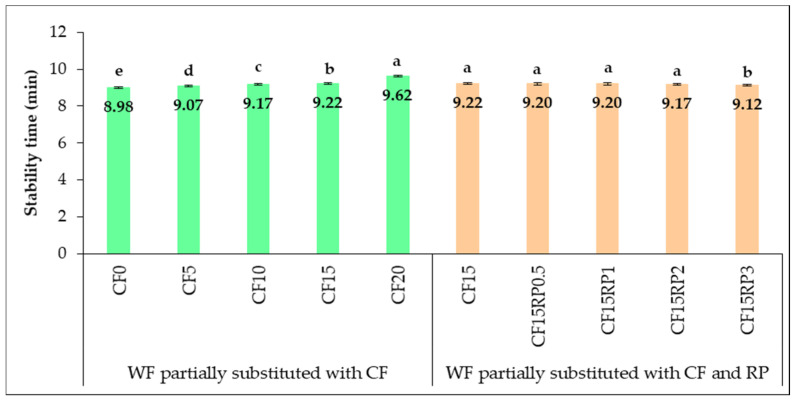
Dough stability time (min) for wheat flour with chestnut flour, and with chestnut flour plus rosehip powder. Group I (CF0, CF5, CF10, CF15, and CF20): wheat flour with 0%, 5%, 10%, 15%, and 20% chestnut flour. Group II (CF15RP0.5, CF15RP1, CF15RP2, and CF15RP3): wheat flour with 15% chestnut flour plus 0.5%, 1%, 2%, and 3% rosehip powder. Data are expressed as mean ± standard deviation of three independent analyses. Different letters above columns indicate significant differences within the same group (one-way ANOVA, *p* < 0.05), while those sharing the same letter are not significantly different (*p* > 0.05).

Figure 6 shows changes in dough stability time with partial WF substitution by CF and CF combined with RP. Dough stability measures resistance to mechanical mixing and indicates gluten strength and overall quality [65,66].

Analysis of the results in Figure 6 reveals an improvement in dough stability, probably resulting from the increase in WF substitution with CF (*p* < 0.05), as mixing time increased from 8.98 min in CF0 to 9.62 min in CF20. These results suggest that CF could improve the dough’s tolerance to mechanical mixing. These results are consistent with those of Moreira et al. [67], who observed high stability (approximately 12.1 min) in CF dough, attributable to its water retention capacity and its stabilizing effect on dough viscosity under thermal stress. The most significant increase in stability was observed between CF10 and CF20, indicating that higher concentrations of CF are associated with a more resistant dough structure, attributable to interactions between CF dietary fibers, residual starch, and the gluten matrix. The addition of RP to CF15 reduced dough stability from 9.22 min (CF15) to 9.12 min for CF15RP3. However, this decrease became statistically significant only at a 3% RP addition, while at 0.5%, 1%, and 2% RP, the reductions were not significant compared with CF15. This observation of weakening is comparable to that reported by Vartolomei and Turtoi [24], attributed to gluten dilution and interference from organic acids and RP fibers. It has been shown that ascorbic acid and phenolic compounds in RP can also induce softening of the dough and reduce its mixing tolerance [68]. Compared to the typical stability times of wheat flour (approximately 5 to 7 min) reported by other authors [65,66,68], the results of the present study are higher, which can be attributed to the use of strong type 650 wheat flour and the rheological effects of CF. CF is a rich source of dietary fiber and sugars and has low levels of lipids that interact with gluten [69]. From a practical standpoint, it has been shown that moderate substitution of wheat flour with CF, up to a rate of 20%, improves dough stability. This improvement in stability can, in turn, lead to improved processability and mixing tolerance in bread and pastry production. However, it should be noted that incorporating more than 2% RP can compromise dough strength. Therefore, careful adjustment is necessary, especially in processes involving prolonged mixing or fermentation.

### 3.3. Phytochemical Content and Antioxidant Activity of WF, CF, and RP

Table 3 shows the total phenolic content (TPC), total flavonoid content (TFC), and antioxidant activity (FRAP and DPPH) of WF, CF, and RP.

The analysis of the WF and enriching agents revealed significant differences in their phytochemical composition and antioxidant potential. The TPC varied markedly among the samples. As shown in Table 3, RP exhibited the highest TPC (2308.47 mg GAE/100 g d.w.), followed by CF (188.27 mg GAE/100 g d.w.), while WF had the lowest value (50.84 mg GAE/100 g d.w.). A similar trend was observed for TFC, with RP showing the highest content (1410.37 mg QE/100 g d.w.), significantly exceeding that of CF (125.27 mg QE/100 g d.w.) and WF (33.78 mg QE/100 g d.w.). These findings align with the previous literature, which consistently highlights rosehip fruits as an exceptional source of phenolic compounds and flavonoids [70,71,72]. For example, Soare et al. [72] reported TPC values ranging from 35.43 to 48.07 mg GAE/g in *Rosa canina* from wild flora, underlining the strong influence of species and environmental conditions on phenolic content.

With regard to antioxidant activity, RP showed the highest performance in both ferric-reducing antioxidant power (FRAP) and DPPH radical scavenging assays, which measure electron-donating and radical-quenching capacities, respectively. The FRAP value for RP was 515.86 µM Fe^2+^/g d.w., substantially higher than CF (23.40 µM Fe^2+^/g d.w.) and WF (2.23 µM Fe^2+^/g d.w.). Similarly, the DPPH radical scavenging activity followed the same pattern: RP recorded 592.85 µM TE/g d.w., compared to CF (29.47 µM TE/g d.w.) and WF (2.84 µM TE/g d.w.). These results are attributed to the high levels of bioactive phenolic and flavonoid compounds in RP, which contribute to antioxidant activity through radical scavenging and reducing mechanisms [31,70]. A correlation between total phenolic content and antioxidant activity in rosehip extracts has also been documented [70].

CF showed strong antioxidant activity and a rich phytochemical profile. Studies have reported that its composition can vary depending on the cultivar, growing region, and processing methods [73]. Our results agree with previous research that highlights *Castanea sativa* as a good source of polyphenols and antioxidants [8]. Additionally, RP is recognized for its considerable antioxidant capacity, which comes from its high levels of polyphenols, flavonoids, and vitamin C [23,29,30]. Regarding WF, it is widely recognized that whole WF contains higher levels of phenolics, flavonoids, and consequently antioxidant activity compared to refined flour. These compounds are the major contributors to the antioxidant potential of cereal-based products [74,75]. The statistically significant differences (*p* < 0.05) among the samples highlight the distinct contributions of each flour/powder to the antioxidant and phytochemical profile of the final bread. These findings suggest that incorporating functional ingredients such as CF and RP can enhance the health-promoting potential of cereal-based foods by enriching their bioactive compounds content.

### 3.4. Phytochemical Content and Antioxidant Activity of Dough and Bread Formulations

The effects of gradual supplementation with CF alone and CF combined with RP on the phytochemical content and antioxidant activity of both dough and finished bread were investigated. The corresponding values for the dough, measured during the bread-making process, are summarized in Table 4.

Supplementation of the dough with CF (0–20%) progressively enhanced its bioactive and antioxidant properties, resulting in 1.45-, 1.46-, 2.57-, and 2.73-fold increases in TPC, TFC, FRAP, and DPPH, respectively, from DCF0 to DCF20. Further enrichment of the 15% CF dough with RP (0.5–3%) enhanced its functionality by providing additional bioactive compounds, leading to 2.06-, 1.96-, 4.45-, and 3.90-fold increases in TPC, TFC, FRAP, and DPPH, respectively, from DCF15 to DCF15RP3. These findings are consistent with previous studies, which have shown that the antioxidant capacity of bread increases with the amount of CF [17]. In a similar manner, enriching WF bread with RP has been shown to significantly enhance its antioxidant activity, total phenolic content, and flavonoid levels [31,72]. It has been reported that adding functional ingredients can increase the phenolic compound content and thereby enhance the antioxidant activity of the dough [76]. However, the native phenolic and flavonoid contents of CF and RP were not fully reflected in the enriched dough samples. Despite their high intrinsic TPC and TFC, the measurable levels in the dough were lower than expected. This can be explained by the strong interactions between phenolic compounds and components of the food matrix, particularly proteins and starch in both WF and CF, which lead to the formation of complexes that reduce the availability of free phenolics. Consequently, the effectiveness of CF and RP enrichment depends not only on the quantity added but also on the extent to which their bioactive compounds remain extractable and bioavailable within the dough matrix [77]. The increases in TPC, TFC, and antioxidant activity in dough fortified with CF and RP indicate potential health benefits, as these compounds neutralize free radicals and improve the functional properties of the bread beyond basic nutrition.

**Figure 7:**
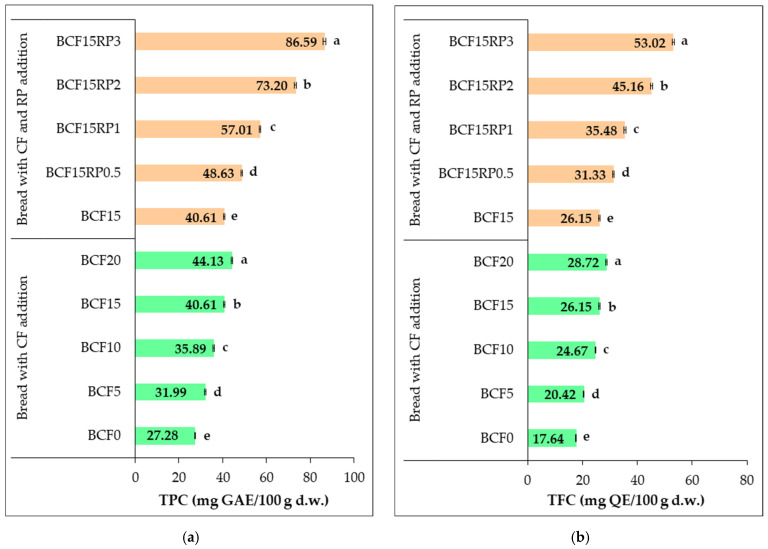
Changes in total phenolic content (TPC) (**a**) and total flavonoid content (TFC) (**b**) in bread with chestnut flour, and with chestnut flour plus rosehip powder. Group I (BCF0, BCF5, BCF10, BCF15, and BCF20): bread from wheat flour with 0%, 5%, 10%, 15%, and 20% chestnut flour. Group II (BCF15RP0.5, BCF15RP1, BCF15RP2, and BCF15RP3): bread from wheat flour with 15% chestnut flour plus 0.5%, 1%, 2%, and 3% rosehip powder. Data are expressed as mean ± standard deviation of three independent analyses. Different letters above columns indicate significant differences within the same group (one-way ANOVA, *p* < 0.05).

The total phenolic content and total flavonoid content of the bread samples are shown in Figure 7.

Increasing the level of CF, and incorporating RP into the 15% CF-substituted bread, progressively enhanced the TPC in the enriched bread formulations. Among the CF-enriched breads, the highest TPC was observed in the sample with 20% CF (BCF20, 44.13 mg GAE/100 g d.w.), representing a 1.62-fold increase compared to the control (BCF0, 27.28 mg GAE/100 g d.w.). This trend highlights the contribution of CF to the TPC of bread through its intrinsic phenolic compounds and aligns with previous reports [78,79]. In the 15% CF bread, the addition of RP further enhanced TPC, increasing from 40.61 mg GAE/100 g d.w. in BCF15 to 86.60 mg GAE/100 g d.w. in BCF15RP3 (2.13-fold vs. BCF15), more than doubling the phenolic content. These results highlight the effectiveness of RP as a rich source of phenolic compounds, significantly boosting the antioxidant potential of bread. Similar findings were reported by Ivanova et al. [31], who observed substantially higher total phenolic and flavonoid levels in wheat flour bread enriched with CF and RP compared to the control. Other studies [80,81,82] also confirm the high content of bioactive compounds in rosehip, particularly polyphenols, vitamin C, and flavonoids, supporting its role in enhancing the antioxidant capacity of bakery products.

A similar pattern was observed for TFC. The control sample (BCF0) exhibited a TFC of 17.64 mg QE/100 g d.w., which progressively increased with the addition of CF, reaching 28.72 mg QE/100 g d.w. in BCF20 (1.63-fold vs. BCF0). The inclusion of RP into the 15% CF sample further increased TFC from 26.15 mg QE/100 g d.w. in BCF15 to 53.02 mg QE/100 g d.w. in BCF15RP3 (2.03-fold vs. BCF15). This two-fold increase suggests that RP not only enhances phenolic content but also substantially enriches the flavonoid profile of the bread. Supporting these findings, Vartolomei and Turtoi [24] reported that adding RP to WF significantly increased both TPC and TFC, attributing this effect to the high polyphenolic content of RP. Another study [2] documented increased levels of phenolics and flavonoids in CF-based products, confirming the presence of bioactive compounds.

Overall, these results demonstrate that both CF and RP positively impact the bioactive profile of bread, with RP showing a more pronounced effect. The dose-dependent and statistically significant (*p* < 0.05) increases in TPC and TFC highlight the contribution of these ingredients to enhancing the bioactive composition of the bread.

**Figure 8:**
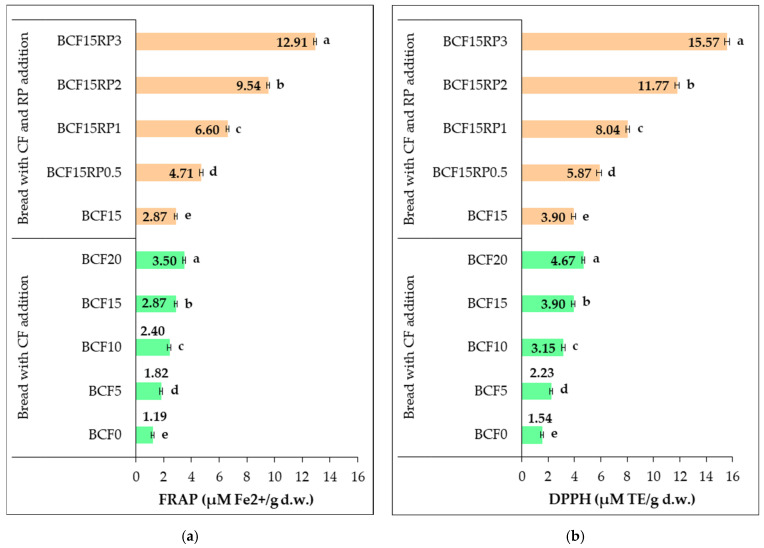
Changes in antioxidant activity measured by FRAP (**a**) and DPPH (**b**) in bread with chestnut flour, and with chestnut flour plus rosehip powder. Group I (BCF0, BCF5, BCF10, BCF15, and BCF20): bread from wheat flour with 0%, 5%, 10%, 15%, and 20% chestnut flour. Group II (BCF15RP0.5, BCF15RP1, BCF15RP2, and BCF15RP3): bread from wheat flour with 15% chestnut flour plus 0.5%, 1%, 2%, and 3% rosehip powder. Data are expressed as mean ± standard deviation of three independent analyses. Different letters above columns indicate significant differences within the same group (one-way ANOVA, *p* < 0.05).

The antioxidant capacity of the bread samples was further evaluated using FRAP (ferric-reducing antioxidant power) and DPPH (2,2-diphenyl-1-picrylhydrazyl) radical scavenging assays, as presented in Figure 8.

Both methods showed statistically significant (*p* < 0.05) increases in antioxidant activity corresponding to higher levels of CF and RP, clearly demonstrating the contribution of these ingredients to the functional enhancement of the bread formulations.

According to the FRAP assay, the control bread (BCF0) had the lowest antioxidant capacity (1.20 µM Fe^2+^/g d.w.). Incremental supplementation with CF increased the FRAP value to 3.50 µM Fe^2+^/g d.w. in BCF20 (2.93-fold). The addition of RP to the 15% CF bread formulation further enhanced FRAP, reaching 12.91 µM Fe^2+^/g d.w. in BCF15RP3 (4.49-fold compared to BCF15). Similarly, DPPH radical scavenging activity increased from 1.54 µM TE/g d.w. in BCF0 to 3.90 µM TE/g d.w. (2.53-fold) in BCF15 and 4.67 µM TE/g d.w. (3.03-fold) in BCF20. RP addition led to a further increase in DPPH values, ranging from 5.87 µM TE/g d.w. (1.50-fold vs. BCF15) in BCF15RP0.5 to 15.57 µM TE/g d.w. (3.99-fold vs. BCF15) in BCF15RP3.

These results demonstrate that RP effectively enhances the radical scavenging capacity of the fortified bread in a dose-dependent and statistically significant (*p* < 0.05) manner.

Our results are consistent with previous studies [4,17,83], which reported enhanced antioxidant capacity in both gluten-free and wheat flour bread supplemented with CF. This effect is attributed to the presence of polyphenols and other antioxidant-active compounds in CF. Similarly, Marciniak-Łukasiak et al. [5] observed a significant improvement in antioxidant activity across all formulations of gluten-free bread enriched with CF compared to the control. Samples enriched with 15% CF and RP exhibited superior antioxidant activity compared to those containing only CF. This is consistent with Chochkov et al. [27], who reported significantly higher radical scavenging capacity in RP-enriched wheat bread, attributed to the high content of bioactive compounds in RP. Similarly, Sanfilippo et al. [28] observed enhanced antioxidant potential in sourdough bread fortified with RP, suggesting its use as a functional additive to improve the health properties of baked products. Additionally, Çürük et al. [22] confirmed that rosehip seed powder has a high total phenolic content, explaining its positive effects on antioxidant activity measured by DPPH and FRAP assays, which supports our findings, particularly in RP-substituted samples.

Overall, both CF and RP notably improve the bioactive profile and antioxidant potential of bread, highlighting their value in the development of functional bakery products. RP, in particular, exerts a stronger effect due to its high content of bioactive compounds, consistent with previous research demonstrating the potent antioxidant properties of rosehip-derived ingredients [58]. Moreover, the synergistic effect observed in formulations containing both CF and RP further supports their potential for developing health-oriented bakery products.

### 3.5. Retention Rate of Phytochemical Content and Antioxidant Activity in Bread Formulations After Baking

**Figure 9:**
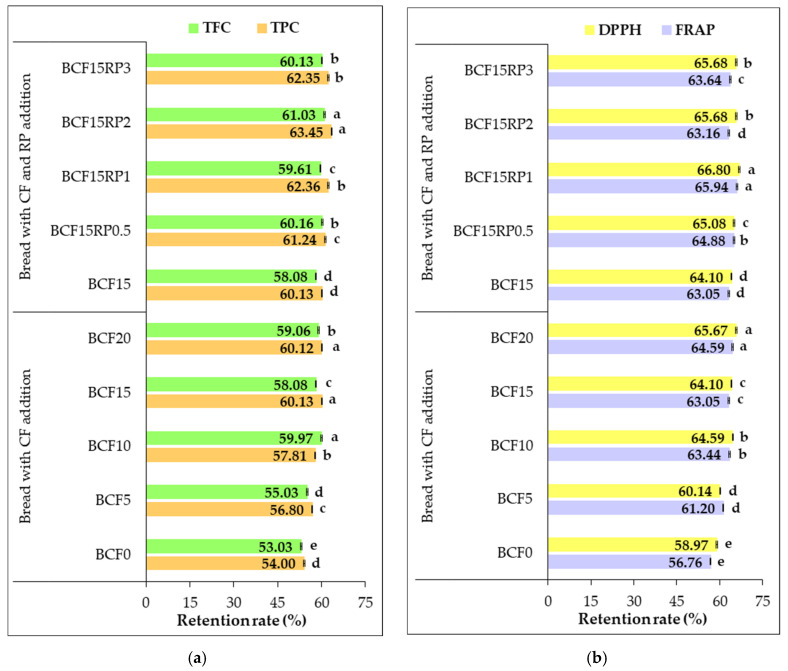
Retention rate (%) of (**a**) total phenolic content (TPC) and total flavonoid content (TFC), and (**b**) antioxidant activity measured by FRAP and DPPH, after baking bread. Group I (BCF0, BCF5, BCF10, BCF15, and BCF20): bread from wheat flour with 0%, 5%, 10%, 15%, and 20% chestnut flour. Group II (BCF15RP0.5, BCF15RP1, BCF15RP2, and BCF15RP3): bread from wheat flour with 15% chestnut flour plus 0.5%, 1%, 2%, and 3% rosehip powder. Data are expressed as mean ± standard deviation of three independent analyses. Different letters above columns indicate significant differences within the same group (one-way ANOVA, *p* < 0.05).

In addition to the close relationship between the functional properties of bread formulations and the levels of CF and RP incorporated, this study evaluates the retention rate of bioactive compounds and antioxidant activity after baking. Retention rate, representing the percentage of TPC, TFC, and antioxidant activity preserved from dough to final bread, highlight losses during baking and assess the stability of these compounds in the finished products. Figure 9 shows the retention rates of TPC, TFC, DPPH, and FRAP for bread formulations enriched with CF alone or combined with RP, indicating that CF enrichment improved retention and RP addition further enhanced preservation, with RP-enriched samples exceeding both control and CF-only formulations across all indicators.

Bread dough formulations and baking significantly affect antioxidant properties, phenolic compound stability, and bioavailability [84,85]. Baking also improves texture and palatability, although heat exposure can cause degradation or structural modification of phenolic compounds [74,86].

Retention rates of TPC in CF-only bread samples ranged from 54.00% to 60.13%, while formulations with RP supplementation showed higher retention, between 61.24% and 63.45%. Similarly, TFC retention increased from 53.03–59.06% in CF-only samples to 59.61–61.03% with RP addition. FRAP values ranged from 56.76–64.59% in CF-only samples and 63.16–65.94% in RP-supplemented formulations. DPPH results followed the same trend, with 58.97–65.67% retention in CF-only and 65.08–66.80% in RP-enriched samples. These results indicate that although baking causes some degradation of bioactive compounds, a substantial proportion is retained, and RP supplementation enhances the stability of functional attributes. The effect was not strictly proportional to RP concentration, showing that complex interactions influence retention. The high content of bioactive compounds in RP, together with synergistic interactions with CF components, enhances antioxidant activity and thermal stability and further supports matrix resilience during baking. Substantial retention rates may be explained by the release of bound phenolics during processing [87] or the formation of antioxidant Maillard reaction products, both contributing to the preservation of antioxidant activity after baking [38]. Matrix interactions between CF and RP components, including complex formation between RP polyphenols and starch/proteins in CF-based doughs, as well as the presence of fiber and organic acids in RP lowering dough pH, further support the higher retention observed. These mechanisms align with the elevated FRAP and DPPH retention rates in RP-enhanced formulations, reaching 65.94% and 66.80% in BCF15RP1, respectively, reflecting preserved or even enhanced electron-donating and radical scavenging potential compared to BCF15.

While all RP-fortified formulations showed elevated retention, the difference between 2% and 3% RP was modest, suggesting a threshold beyond which additional RP does not proportionally enhance antioxidant preservation. This underlines the importance of carefully managing RP levels in functional product development.

Overall, the results demonstrate functional synergy between CF and RP, enhancing both the nutritional profile and the resilience of key phytochemicals during baking.

The retention of these compounds indicates that CF- and RP-enriched bread formulations can act as effective vehicles for delivering dietary antioxidants. Their preservation after baking supports the potential of these ingredients for developing functional bakery products with improved nutritional value and health-promoting properties. Understanding the retention of TPC and TFC is essential for designing formulations with enhanced functional qualities, and investigating how processing steps, particularly baking, affect compound stability ensures their presence in the final product. Future studies should characterize individual polyphenols and investigate their stability during baking and in multi-ingredient matrices to clarify retention mechanisms and implications for storage and sensory properties.

### 3.6. Physical Characteristics of Bread Formulations

The physical characteristics, including elasticity, porosity, and height-to-diameter (H/D) ratio, of the bread formulations are shown in Table 5.

The evaluation of the physical characteristics of the bread formulations showed statistically significant differences (*p* < 0.05) between the control sample (BCF0) and the samples containing only CF, as well as between the 15% CF-fortified sample (BCF15) and the samples with 15% CF and additional RP incorporation.

The BCF0 sample exhibited the highest values for elasticity (90.95%), porosity (77.44%), and the height-to-diameter (H/D) ratio (0.58), compared to the CF-enriched samples, which showed lower values for these parameters. Elasticity decreased progressively from 87.22% in BCF5 to 77.43% in BCF20. Similarly, in RP-enriched bread, elasticity declined from 78.15% in BCF15RP0.5 to 72.70% in BCF15RP3, compared to 80.97% in the corresponding 15% CF-enriched sample without RP.

Porosity also showed a decreasing trend proportional to the increasing substitution levels of WF with CF and the combination of CF and RP. The H/D ratio showed a progressive decrease with increasing levels of WF substitution by CF and RP. In CF-enriched samples, the H/D ratio gradually declined from 0.559 (BCF5) to 0.525 (BCF20). Similarly, in RP-enriched formulations, the H/D ratio decreased from 0.53 (BCF15RP0.5) to 0.49 (BCF15RP3), compared to 0.54 in the corresponding 15% CF-enriched sample without RP.

The addition of CF and RP, due to their high dietary fiber content, reduces bread elasticity and porosity, as the fibers interfere with the formation and stability of the gluten network, which is responsible for the rheological properties of the dough and crumb structure. Increasing the proportion of these components intensifies this negative effect, diminishing the ability of the bread to retain fermentation gases and maintain a porous, elastic structure [61].

The H/D ratio showed a progressive decrease with increasing levels of WF substitution by CF and RP. In CF-enriched samples, the H/D ratio gradually declined from 0.56 (BCF5) to 0.53 (BCF20). Similarly, in RP-enriched formulations, the H/D ratio decreased from 0.53 (BCF15RP0.5) to 0.493 (BCF15RP3), compared to 0.54 in the corresponding 15% CF-enriched sample without RP.

The results showed a progressive reduction in physical characteristic values with increasing levels of WF substitution by CF, and with additional increasing levels of RP in the sample containing 15% CF, indicating a significant impact on the doughs’ viscoelastic properties. However, moderate substitution levels still yielded physical characteristics within acceptable limits for bread [33,44,88]. These findings align with the previous literature, showing that fiber-rich alternative flours affect gluten network structure but can be used in moderate amounts for developing functional bread [61].

## 4. Conclusions

This study offers practical insights into using chestnut flour (CF) and rosehip powder (RP) as partial wheat flour substitutes to enhance bread nutritional and functional properties. Partial substitution of wheat flour with CF improved dough stability, mixing tolerance, and starch behavior at 5–10%, while higher levels (15–20%) slightly reduced protein strength and starch gelatinization, without compromising dough performance. Incorporation of CF (5–15%) increased phytochemical content and antioxidant activity in dough and bread, while adding RP (0.5–2%) to the 15% CF formulation further enhanced these effects and starch performance, without causing additional weakening of the dough structure. Water absorption remained largely stable, while starch retrogradation decreased with CF but showed a slight increase with RP. Baking reduced bioactive compounds, with TPC, TFC, FRAP, and DPPH retaining 59–66% at the highest CF-substitution level and 61–67% in RP-enriched bread. Bread structure was preserved, with only minor decreases in elasticity, porosity, and height-to-diameter ratio at higher substitution levels. The combination of 15% CF with 2% RP provided a balanced improvement in nutritional quality, bioactive content, and technological properties, maintaining adequate dough and bread performance and demonstrating the potential of CF- and RP-enriched bread as a functional bakery product. This study highlights how these unconventional ingredients can be successfully incorporated into widely consumed bread, improving both nutritional and bioactive profiles and providing a model for developing novel functional bakery products. Overall, CF and RP represent valuable ingredients for producing breads with enhanced health-promoting properties. The measured parameters could be integrated into a systematized mechanism linking ingredient composition, dough development, starch gelatinization, amylase activity, and bread quality. Ingredient composition and RP addition modulate water absorption, gluten network formation, viscosity, and retrogradation, synergistically shaping dough properties and final bread quality. Future research should address polyphenol stability during baking, sensory evaluation, and additional Mixolab parameters to better understand dough behavior and starch retrogradation in relation to bread shelf life.

## Figures and Tables

**Table 1:** Proximate composition and energy value of wheat flour, chestnut flour, and rosehip powder.

Samples	Chemical Parameters						Energy Value (kcal/100 g)
	Moisture (%)	Fat (%)	Protein (%)	Fiber (%)	Ash (%)	CRB (%)	
WF	12.74 ± 0.03 ^a^	1.56 ± 0.02 ^c^	12.89 ± 0.02 ^a^	3.41 ± 0.04 ^c^	0.64 ± 0.01 ^c^	72.17 ± 0.03 ^c^	354.28 ± 0.04 ^c^
CF	11.52 ± 0.02 ^b^	3.91 ± 0.03 ^b^	7.79 ± 0.04 ^b^	7.25 ± 0.06 ^b^	3.01 ± 0.02 ^b^	73.77 ± 0.04 ^b^	361.43 ± 0.05 ^b^
RP	7.26 ± 0.03 ^c^	4.87 ± 0.03 ^a^	6.49 ± 0.05 ^c^	56.09 ± 0.02 ^a^	4.49 ± 0.01 ^a^	76.89 ± 0.04 ^a^	377.35 ± 0.05 ^a^

CRB: carbohydrates; WF: wheat flour; CF: chestnut flour; RP: rosehip powder. Data are expressed as mean ± standard deviation of three independent analyses. Values marked with different letters within the same column are significantly different (one-way ANOVA, *p* < 0.05).

**Table 2:** Proximate composition and energy value of bread formulations with chestnut flour, and with chestnut flour plus rosehip powder.

Samples	Chemical Parameters						Energy Value (kcal/100 g)
	Moisture (%)	Fat (%)	Protein (%)	Fiber (%)	Ash (%)	CRB (%)	
BCF0	37.72 ± 0.03 ^a^	1.15 ± 0.02 ^e^	9.55 ± 0.03 ^a^	2.53 ± 0.06 ^e^	0.48 ± 0.02 ^e^	51.10 ± 0.03 ^a^	252.95 ± 0.05 ^e^
BCF5	37.68 ± 0.03 ^a^	1.24 ± 0.04 ^d^	9.36 ± 0.04 ^b^	2.67 ± 0.05 ^d^	0.56 ± 0.05 ^d^	51.16 ± 0.04 ^a^	253.24 ± 0.04 ^d^
BCF10	37.65 ± 0.03 ^a^	1.33 ± 0.03 ^c^	9.17 ± 0.03 ^c^	2.81 ± 0.06 ^c^	0.65 ± 0.03 ^c^	51.20 ± 0.03 ^a^	253.45 ± 0.05 ^c^
BCF15	37.62 ± 0.03 ^a^	1.41 ± 0.02 ^b^	8.98 ± 0.04 ^d^	2.95 ± 0.04 ^b^	0.74 ± 0.04 ^b^	51.25 ± 0.04 ^a^	253.61 ± 0.04 ^b^
BCF20	37.59 ± 0.02 ^a^	1.50 ± 0.04 ^a^	8.79 ± 0.03 ^e^	3.09 ± 0.05 ^a^	0.83 ± 0.02 ^a^	51.29 ± 0.04 ^a^	253.82 ± 0.03 ^a^
BCF15	37.62 ± 0.02 ^a^	1.41 ± 0.02 ^a^	8.98 ± 0.04 ^a^	2.95 ± 0.06 ^e^	0.74 ± 0.04 ^a^	51.25 ± 0.04 ^a^	253.61 ± 0.04 ^a^
BCF15RP0.5	37.62 ± 0.02 ^a^	1.43 ± 0.02 ^a^	8.96 ± 0.05 ^a^	3.14 ± 0.05 ^d^	0.75 ± 0.04 ^a^	51.24 ± 0.05 ^a^	253.67 ± 0.05 ^a^
BCF15RP1	37.61 ± 0.02 ^a^	1.44 ± 0.03 ^a^	8.93 ± 0.06 ^a^	3.33 ± 0.03 ^c^	0.77 ± 0.03 ^a^	51.25 ± 0.03 ^a^	253.68 ± 0.04 ^a^
BCF15RP2	37.60 ± 0.02 ^a^	1.46 ± 0.06 ^a^	8.89 ± 0.03 ^a^	3.72 ± 0.04 ^b^	0.80 ± 0.03 ^a^	51.25 ± 0.05 ^a^	253.71 ± 0.06 ^a^
BCF15RP3	37.59 ± 0.02 ^a^	1.49 ± 0.03 ^a^	8.84 ± 0.03 ^a^	4.10 ± 0.06 ^a^	0.82 ± 0.03 ^a^	51.26 ± 0.04 ^a^	253.81 ± 0.05 ^a^

CRB: carbohydrates; Group I (BCF0, BCF5, BCF10, BCF15, and BCF20): bread from wheat flour with 0%, 5%, 10%, 15%, and 20% chestnut flour. Group II (BCF15RP0.5, BCF15RP1, BCF15RP2, and BCF15RP3): bread from wheat flour with 15% chestnut flour plus 0.5%, 1%, 2%, and 3% rosehip powder. Data are expressed as mean ± standard deviation of three independent analyses. Values marked with different letters within the same column are significantly different (one-way ANOVA, *p* < 0.05), while those with the same letter are not significantly different (*p* > 0.05).

**Table 3:** Total phenolic content, total flavonoid content, and antioxidant activity of wheat flour, chestnut flour and rosehip powder.

Sample	FRAP (µM Fe^2+^/g d.w.)	DPPH (µM TE/g d.w.)	TPC (mg GAE/100 g d.w.)	TFC (mg QE/100 g d.w.)
WF	2.23 ± 0.06 ^c^	2.84 ± 0.07 ^c^	50.84 ± 0.40 ^c^	33.78 ± 0.27 ^c^
CF	23.40 ± 1.26 ^b^	29.47 ± 1.43 ^b^	188.27 ± 1.47 ^b^	125.27 ± 1.69 ^b^
RP	515.86 ± 1.57 ^a^	592.85 ± 1.63 ^a^	2308.47 ± 1.96 ^a^	1410.37 ± 1.79 ^a^

WF: wheat flour; CF: chestnut flour; RP: rosehip powder; Values reported are the mean of three independent analyses ± standard deviation (SD). Data are expressed as mean ± standard deviation from three independent analyses. Values marked with different letters within the same column are significantly different (one-way ANOVA, *p* < 0.05).

**Table 4:** Antioxidant activity (FRAP and DPPH), total phenolic content (TPC), and total flavonoid content (TFC) of dough during the bread-making process.

Sample	FRAP (µM Fe^2+^/g d.w.)	DPPH (µM TE/g d.w.)	TPC (mg GAE/100 g d.w.)	TFC (mg QE/100 g d.w.)
DCF0	2.11 ± 0.07 ^e^	2.61 ± 0.65 ^e^	50.52 ± 0.49 ^e^	33.27 ± 0.32 ^e^
DCF5	2.97 ± 0.11 ^d^	3.72 ± 0.12 ^d^	56.32 ± 0.62 ^d^	37.10 ± 0.60 ^d^
DCF10	3.79 ± 0.11 ^c^	4.87 ± 0.131 ^c^	62.08 ± 1.004 ^c^	41.15 ± 0.89 ^c^
DCF15	4.56 ± 0.09 ^b^	6.09 ± 0.10 ^b^	67.54 ± 1.11 ^b^	45.02 ± 1.01 ^b^
DCF20	5.41 ± 0.13 ^a^	7.11 ± 0.13 ^a^	73.40 ± 1.22 ^a^	48.63 ± 1.24 ^a^
DCF15	4.56 ± 0.087 ^e^	6.085 ± 0.10 ^e^	67.54 ± 1.11 ^e^	45.02 ± 1.01 ^e^
DCF15RP0.5	7.26 ± 0.079 ^d^	9.018 ± 0.09 ^d^	79.41 ± 1.08 ^d^	52.07 ± 0.95 ^d^
DCF15RP1	10.02 ± 0.13 ^c^	12.032 ± 0.13 ^c^	91.42 ± 1.10 ^c^	59.51 ± 1.10 ^c^
DCF15RP2	15.11 ± 0.15 ^b^	17.914 ± 0.14 ^b^	115.37 ± 1.22 ^b^	74.01 ± 1.21 ^b^
DCF15RP3	20.28 ± 0.16 ^a^	23.707 ± 0.17 ^a^	138.89 ± 1.30 ^a^	88.18 ± 1.16 ^a^

Group I (DCF0, DCF5, DCF10, DCF15, and DCF20): dough from wheat flour with 0%, 5%, 10%, 15%, and 20% chestnut flour. Group II (DCF15RP0.5, DCF15RP1, DCF15RP2, and DCF15RP3): dough from wheat flour with 15% chestnut flour plus 0.5%, 1%, 2%, and 3% rosehip powder. Data are expressed as mean ± standard deviation of three independent analyses. Values marked with different letters within the same column are significantly different (one-way ANOVA, *p* < 0.05).

**Table 5:** Physical characteristics of bread with chestnut flour and chestnut flour plus rosehip powder.

Samples	Elasticity (%)	Porosity (%)	Height/Diameter (H/D)
BCF0	90.95 ± 0.20 ^a^	77.44 ± 0.14 ^a^	0.58 ± 0.008 ^a^
BCF5	87.22 ± 0.19 ^b^	75.97 ± 0.11 ^b^	0.56 ± 0.005 ^b^
BCF10	84.55 ± 0.16 ^c^	74.89 ± 0.13 ^c^	0.55 ± 0.003 ^c^
BCF15	80.97 ± 0.14 ^d^	73.70 ± 0.12 ^d^	0.54 ± 0.004 ^d^
BCF20	77.43 ± 0.12 ^e^	72.32 ± 0.10 ^e^	0.53 ± 0.003 ^e^
BCF15	80.97 ± 0.16 ^a^	73.70 ± 0.12 ^a^	0.54 ± 0.004 ^a^
BCF15RP0.5	78.15 ± 0.14 ^b^	73.06 ± 0.11 ^b^	0.53 ± 0.005 ^a^
BCF15RP1	76.88 ± 0.12 ^c^	72.39 ± 0.10 ^c^	0.52 ± 0.006 ^a^
BCF15RP2	74.32 ± 0.13 ^d^	71.24 ± 0.12 ^d^	0.50 ± 0.003 ^b^
BCF15RP3	72.70 ± 0.11 ^e^	70.16 ± 0.11 ^e^	0.49 ± 0.004 ^c^

Group I (BCF0, BCF5, BCF10, BCF15, and BCF20): bread from wheat flour with 0%, 5%, 10%, 15%, and 20% chestnut flour. Group II (BCF15RP0.5, BCF15RP1, BCF15RP2, and BCF15RP3): bread from wheat flour with 15% chestnut flour plus 0.5%, 1%, 2%, and 3% rosehip powder. Data are expressed as mean ± standard deviation of three independent analyses. Means are expressed with two decimals; standard deviations are expressed with three decimals when below 0.01 to preserve statistical significance. Values marked with different letters within the same column are significantly different (one-way ANOVA, *p* < 0.05), while those with the same letter are not significantly different (*p* > 0.05).

## Data Availability

Original data and contributions presented in this study are provided in the article and are accessible at the University of Life Sciences “King Mihai I” from Timisoara.
